# Role of maternal preconception nutrition on offspring growth and risk of stunting across the first 1000 days in Vietnam: A prospective cohort study

**DOI:** 10.1371/journal.pone.0203201

**Published:** 2018-08-30

**Authors:** Melissa F. Young, Phuong Hong Nguyen, Ines Gonzalez Casanova, O. Yaw Addo, Lan Mai Tran, Son Nguyen, Reynaldo Martorell, Usha Ramakrishnan

**Affiliations:** 1 Hubert Department of Global Health, Rollins School of Public Health, Emory University, Atlanta, Georgia, United States of America; 2 International Food Policy Research Institute, Washington, District of Columbia, United States of America; 3 Thai Nguyen University of Pharmacy and Medicine, Thai Nguyen, Vietnam; The University of Manchester, UNITED KINGDOM

## Abstract

Growing evidence supports the role of preconception maternal nutritional status (PMNS) on birth outcomes; however, evidence of relationships with child growth are limited. We examined associations between PMNS (height, weight and body mass index- BMI) and offspring growth during the first 1000 days. We used prospective cohort data from a randomized-controlled trial of preconception micronutrient supplementation in Vietnam, PRECONCEPT (n = 1409). Poisson regression models were used to examine associations between PMNS and risk of offspring stunting (<-2 HAZ) at 2 years. We used path analytic models to examine associations with PMNS on fetal growth (ultrasound measurements) and offspring HAZ at birth and 2 years. All models were adjusted for child age, sex, gestational weight gain, education, socioeconomic status and treatment group. A third of women had a preconception height < 150cm or weight < 43 kg. Women with preconception height < 150 cm or a weight < 43 kg were at increased risk of having a stunted child at 2 years (incident risk ratio IRR: 1.85, 95% CI 1.51–2.28; IRR 1.35, 95% CI 1.10–1.65, respectively). While the traditional low BMI cut-off (< 18.5 kg/m^2^) was not significant, lower BMI cut-offs (< 17.5 kg/m^2^ or < 18.0 kg/m^2^) were significantly associated with 1.3 times increased risk of child stunting. In path models, PMNS were positively associated with fetal growth (ultrasound measurements) and offspring HAZ at birth and 2 years. For each 1 standard deviation (SD) increase in maternal height and weight, offspring HAZ at 2 years increased by 0.30 SD and 0.23 SD, respectively. In conclusion, PMNS influences both offspring linear growth and risk of stunting across the first 1000 days. These findings underscore the importance of expanding the scope of current policies and strategies to include the preconception period in order to reduce child stunting.

## Introduction

Maternal nutrition plays a key role in fetal growth, infant health and survival as well as long-term child health and development [1]. During the first half of the critical 1000 days period (conception to 6 months) the mother is the sole source of nutrition for the developing child; first in utero and then during the first 6 months of life when exclusive breastfeeding is recommended [2]. The Lancet 2013 nutrition series has identified maternal undernutrition during pregnancy as a major determinant of poor fetal growth and child stunting [1]. Women with a height <145 cm or BMI <18.5 kg/m^2^ during early pregnancy are at greater risk of delivering a small for gestational age (SGA) infant (odds ratio 2.12; 95% CI 1.88–2.39; and 1.60; 95% CI 1.45–1.77, respectively) [1]. Furthermore, SGA, a marker for fetal growth restriction, is associated with an increased risk of child morbidity and mortality and is estimated to contribute to approximately 20% of stunting cases globally [3–5]. Stunting is in turn associated with increased risk of child morbidity and mortality, poor cognition, lower school performance and human capital measures such as decreased earning potential, adult stature and increased risk of chronic disease later in life [1,6]. In many low to middle income countries, rapid linear growth failure occurs in the first 1000 days of life [6], and although progress has been made in reducing the burden of stunting in some settings [7], nearly 1 out of 4 children worldwide are stunted. Given the short and long term consequences of linear growth retardation during the first 1000 days of life, the prevention of stunting is a key global priority [8].

A focus on women’s nutrition during the first 1000 days is an important first step [2]; however, questions remains to whether providing nutrition interventions in pregnancy is early enough to prevent child growth failure. Emerging research has shown the importance of preconception maternal nutritional status (PMNS) for improving birth outcomes [9–12]. However, compared to data on the pregnancy period, data that allow us to accurately estimate the influence of maternal nutritional status during the preconception period on early child growth are sparse [11, 13–18]. In prior systematic reviews, pre pregnancy short stature, underweight and overweight were associated with giving birth to a preterm or SGA baby, but there were few well-designed studies [9, 11, 19]. Prospectively collected preconception anthropometric data are rare and studies often rely on maternal recall or measures obtained during pregnancy [11]. Further, most studies have focused on birth outcomes, and there are key gaps in our knowledge on the influence of maternal preconception nutritional status on child linear growth.

We have previously reported that preconception nutrition has a similar and independent influence on birth outcomes compared to maternal nutrition during pregnancy using prospective data from a cohort of Vietnamese women [12]. A one standard deviation increase in preconception weight was associated with a 283 g (95% CI: 279–286) increase in birthweight and women with a preconception weight less than 43 kg were nearly 3 times more likely to give birth to a SGA or low birthweight infant [12]. We have since successfully followed up the PRECONCEPT birth cohort to age 2 years, and now have the unique opportunity to use these data to examine associations between preconception maternal nutritional status and offspring linear growth across the first 1000 days.

## Methods

### Data sources and study population

**Fig 1:**
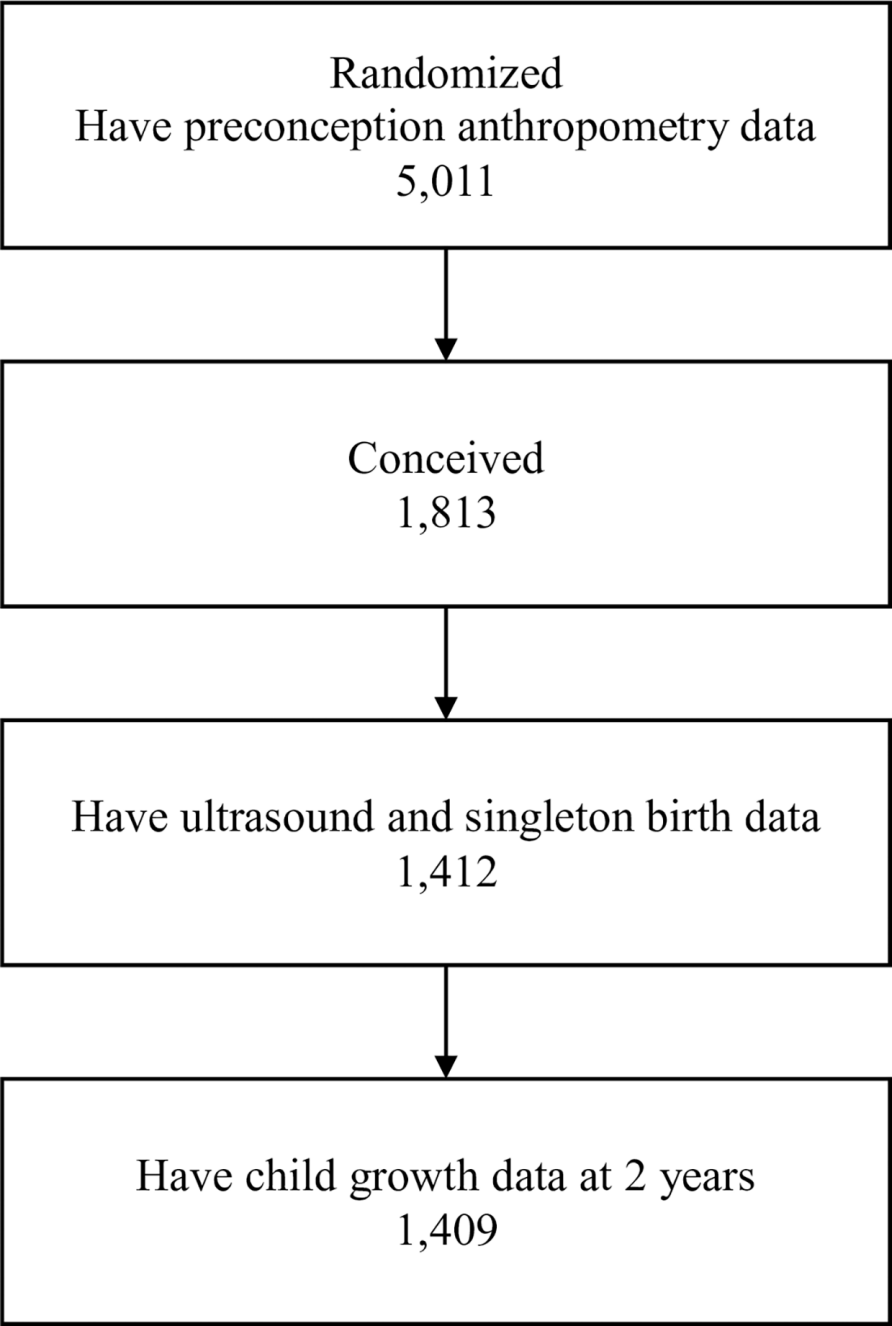
Flow diagram of participant progress throughout the study. Flow diagram of participant enrollment and follow-up of mother-child pairs.

This study uses data obtained from a randomized controlled trial (PRECONCEPT study), which evaluated the effects of preconception micronutrient supplementation on maternal and child health outcomes (*identification number NCT01665378*) [20]. The parent study was approved by the Ethical Committee of the Institute of Social and Medicine Studies in Hanoi, Vietnam and Emory University's Institutional Review Board in Atlanta, Georgia, USA. Written informed consent was obtained from all study participants following approved procedures. This study is reported as per STROBE guidelines (S1 Fig) [21]. There were 5,011 eligible women assigned randomly to one of three pre-pregnancy groups to receive weekly supplements containing either: 1) 2800 μg folic acid (FA; control); 2) 60 mg iron and 2800 μg FA (IFA); or 3) multiple micronutrients (MM) containing the same amount of IFA. Women were followed prospectively to identify pregnancies and evaluate birth outcomes; 1,813 women conceived between 2012–2014 and 1,599 had live births (1579 singleton births, 10 twins). In the second phase, live births were followed to age 2 years. The current analysis includes 1,409 women (**Fig 1**) who met the following inclusion criteria: delivered singleton live infants with available data on maternal preconception height/weight, at least one ultrasound measurement before 30 weeks, and offspring length at birth and at 2 years.

### Outcome measurements

Offspring growth during the first 1000 days was measured by ultrasound measurements for fetal growth and child length at birth and at 2 years of age. Fetal measurements including head circumference (HC), biparietal diameter (BPD), abdominal circumference (AC) and femur length (FL) were obtained during routine prenatal care visits at commune health clinics by trained obstetricians, using real-time ultrasound on a portable machine (Prosound 2, Hitachi Aloka, Japan). Details of the ultrasound examination techniques used are provided elsewhere [22]. Duplicate measures were obtained from separate scans and the averages were converted to Z-scores using the reference values from the INTERGROWTH-21st Project [23].

Child weight and length at birth and at 2 years was collected by trained and standardized field staff using standard methods [24]. Child weight was measured using a UNICEF Beam type scale and recumbent length was measured with collapsible length boards, which were precise to 1 mm. The average of duplicate length measurements was then converted into height-for-age Z-scores (HAZ) according to 2006 WHO child growth standards [25]. Stunting was defined as HAZ below -2 Z-score.

### Predictor variables

Maternal anthropometric measurements were obtained by trained field staff using standard procedures [24, 26] at the time of recruitment (preconception) in Community Health Centers. Preconception weight was measured using calibrated electronic Seca scales to the nearest 0.10 kg. Height was measured in the standing position using a portable stadiometer, measured to the nearest centimeter. All measurements were taken twice and averaged with strict adherence to the measuring techniques and recording procedures. Body mass index (BMI, kg/m^2^) was calculated and categorized as underweight (<18.5), normal (18.5–23) or overweight (≥ 23) using cutoff values recommended to identify high risk individuals in Asians [27]. We also conducted sensitivity analyses using different BMI cut-off values, namely <17, <17.5 and <18 to examine the degree of association with child growth. Maternal weight and height were converted to internal Z-scores for study population to facilitate comparisons of the relative strength of relationships with fetal and child outcomes.

### Confounders

Several factors at maternal, child and household levels were also measured. At the maternal level, we included age, education (completion of primary, middle, high school, or college and higher) and parity. Gestational weight gain was calculated from maternal weight measured at delivery and pre-pregnancy weight. At the child level, we included age, sex and preterm birth and Small for gestational age (SGA). Gestational age was based on the date of last menstrual period that was obtained prospectively by village health workers (VHW) during their biweekly home visits [13]. Preterm birth was defined as a birth occurring before 37 completed weeks of pregnancy and SGA was defined as a birthweight below the 10th percentile for gestational age based on the international newborn standards from the INTERGROWTH-21st Project [24].

Household characteristics included socio-economic status (SES) that was calculated using a principal components analysis of assets and services, including house and land ownership, housing quality, access to services (electricity, gas, water, and sanitation services), and household assets (productive assets, durable goods, animals, and livestock). The first component derived from component scores were used to divide household SES into quartiles [28, 29].

### Statistical analysis

Normality of the continuous outcome variables was assessed using the Kolmogorov-Smirnov test. Descriptive analyses (means, standard deviations, percent) were used to report characteristics of the study population. We used multivariate poisson regression models with robust standard errors to examine associations between PMNS indicators and incident risk ratios (IRR) of stunting at 2 years of age, controlling for various potential confounding variables as mentioned above. We conducted a path analysis to assess the potential mediating effect of fetal growth (ultrasound measurements: HC, BP, AC, and FL) and offspring HAZ at birth on the relationships between PMNS and offspring growth at 2 years [30]. Path analytic models allowed us to simultaneously estimate of all regression equations identified in a model and to examine both the direct effects of PMNS on child growth at 2 years as well as the indirect effects mediated through fetal growth and birth size. The indirect effect was calculated as the product of the unstandardized regression coefficients for each path, using the Sobel test. Total effect of PMNS on child HAZ at 2 years is the sum of indirect and direct effects. All models were adjusted for child age and sex, gestational weight gain, maternal education, socioeconomic status, treatment group and time from maternal enrollment in study to conception. All data analyses were performed using STATA version 13 [31]. Results were considered significant when p <0.05.

## Results

The mean age of mothers was 26 years. More than half of the mothers had completed middle school (54.5%), and 37% had completed high school or higher. Of the 1,409 women included in the study, a third of the women had a prepregnancy BMI <18.5 kg/m^2^, height <150cm or weight less than 43 kg (**Table 1**). The average BMI, height and weight for women were 19.6 kg/m^2^, 152.6 cm, 45.8 kg, respectively. Among the women who became pregnant, the average time from enrollment to conception was 31 weeks. More than two thirds of women had gestational weight gain below the Institute of Medicine (IOM) recommendation [32]. At birth, nearly 12% of children were SGA and 9.5% were preterm. At age 2 y, the prevalence of stunting was 22.2%.

**Table 1:** Maternal and newborn characteristics (n = 1409)^1^.

Characteristic	Mean ± SD or %
**Maternal Indicators**	
Age at baseline (y)	25.8 ± 4.3
Education (%)	
Primary school	8.2
Middle school	54.5
High school	25.8
College or higher	11.6
Primiparous (%)	5.5
Pre-pregnancy weight (kg)	45.8 ± 5.4
Pre-pregnancy weight <43 kg (%)	32.2
Height (m)	152.6 ± 5.1
Height <1.50 (%)	30.3
Pre-pregnancy BMI (kg/m2)	19.6 ± 2.0
BMI < 17.0 (%)	6.7
BMI < 17.5 (%)	12.4
BMI < 18.0 (%)	20.8
BMI < 18.5 (%)	30.3
BMI > 23 (%)	5.9
Gestational Weight gain (kg)	10.0 ± 3.9
Gained below IOM recommendation^2^ (%)	69.2
Gained at IOM recommendation (%)	25.3
Gained above IOM recommendation (%)	5.5
**Fetal Indicators**	
HC Z-score	-1.3 ±1.3
BP Z-score	-1.4 ± 1.2
AC Z-score	-1.2 ± 1.3
FL Z-score	-0.3 ±1.4
**Child Indicators**	
Female (%)	49.7
Birth weight (g)	3080 ± 439.0
Birth length (cm)	49.0 ± 3.0
Preterm birth (%)	9.4
SGA (%)	11.8
HAZ at 2 years	-1.26 ± 0.91
Stunting at 2 years (%)	22.2

^1^Acronyms: AC, abdomen circumference, BP, Biparietal diameter; BMI, body mass index; FL, femoral length; HAZ, height for age Z-score; HC, head circumference, IOM, Institute of Medicine; SGA, small for gestational age based on INTERGROWTH-21^st^ project.

^2^Currently in Vietnam there are no local weight gain recommendations, thus we compared gestational weight gain in relation to Institute of Medicine (IOM) recommendations to define those above or below IOM recommendation [32].

In adjusted multivariate poisson regression models, measures of long-term maternal preconception nutrition and health (maternal height < 150 cm) and current maternal preconception nutritional status (maternal BMI/ weight) were associated with increased risk of child stunting at 2 years of age (**Table 2**). To examine the role of different cut-offs to define low maternal preconception status five models were developed using BMI gradations <18.5 kg/m^2^, <18.0 kg/m^2^, <17.5 kg/m^2^, <17.0 kg/m^2^ as well as using the lowest tertile for maternal weight in our population (< 43 kg). Maternal preconception BMI of <18.0 kg/m^2^, <17.5 kg/m^2^ and preconception weight < 43 kg were all significantly associated with increased risk of child stunting; however, the traditional underweight category of <18.5 kg/m^2^ and the lowest BMI cut-off of <17.0 kg/m^2^ were not significant. In each of the models, preconception height <150 cm was associated with a 2-fold increase in risk (after adjusting for either maternal BMI or weight, respectively). Likewise, in multivariate analyses continuous measures using maternal preconception height z-scores and weight z-scores were significant predictors of child HAZ at 2 years of age (data not shown). There was no evidence of multicollinearity in models.

**Table 2:** Risk of child stunting at 2 years of age by maternal preconception nutritional status.

	Model 1 BMI <17 kg/m^2^	Model 2 BMI <17.5 kg/m^2^	Model 3 BMI <18 kg/m^2^	Model 4 BMI <18.5 kg/m^2^	Model 5 Weight < 43 kg
**Stunting**	IRR (95% CI)	IRR (95% CI)	IRR (95% CI)	IRR (95% CI)	IRR (95% CI)
Mother’s height < 1.5 m	2.07*** (1.70, 2.52)	2.09*** (1.72, 2.54)	2.10*** (1.73, 2.56)	2.08*** (1.71, 2.53)	1.85*** (1.51, 2.28)
Mother’s BMI	1.19 (0.84, 1.68)	1.33* (1.03, 1.72)	1.39** (1.12, 1.72)	1.21+ (0.99, 1.48)	—-
Mother’s weight < 43 kg	—-	—-	—-	—-	1.35** (1.10, 1.65)
Weight gain < IOM recommendation	1.27+ (1.00, 1.63)	1.27+ (0.99, 1.61)	1.26+ (0.99, 1.61)	1.26+ (0.99, 1.61)	1.28* (1.00, 1.63)
Child’s age	0.86* (0.75, 1.00)	0.86* (0.75, 0.99)	0.86* (0.74, 0.99)	0.86* (0.75, 1.00)	0.87+ (0.76, 1.00)
Child as male	1.29* (1.06, 1.57)	1.28* (1.06, 1.56)	1.29* (1.06, 1.57)	1.29* (1.06, 1.57)	1.31** (1.08, 1.59)
Mother’s education (college or higher as reference)					
Primary school	2.30* (1.26, 4.18)	2.31** (1.27, 4.19)	2.30** (1.27, 4.16)	2.29** (1.26, 4.18)	2.29** (1.26, 4.17)
Secondary school	2.14** (1.26, 3.65)	2.11** (1.23, 3.59)	2.09** (1.23, 3.55)	2.12** (1.24, 3.62)	2.12** (1.24, 3.63)
High school	1.70+ (0.98, 2.95)	1.67+ (0.96, 2.89)	1.64+ (0.95, 2.83)	1.69+ (0.97, 2.93)	1.69+ (0.97, 2.94)
Household economic status (highest as reference)					
Lowest	1.41+ (0.96, 2.07)	1.39+ (0.95, 2.05)	1.41+ (0.96, 2.06)	1.41+ (0.96, 2.08)	1.40+ (0.95, 2.06)
Low	1.18 (0.80, 1.74)	1.18 (0.80, 1.75)	1.20 (0.81, 1.77)	1.19 (0.80, 1.75)	1.18 (0.80, 1.74)
Middle	1.25 (0.85, 1.84)	1.24 (0.84, 1.82)	1.25 (0.85, 1.83)	1.26 (0.86, 1.85)	1.26 (0.86, 1.85)
High	1.14 (0.77, 1.67)	1.13 (0.77, 1.66)	1.14 (0.78, 1.67)	1.14 (0.78, 1.68)	1.15 (0.78, 1.68)
Treatment groups (iron as reference)					
Multiple micronutrient	0.84 (0.67, 1.06)	0.85 (0.67, 1.08)	0.85 (0.68, 1.08)	0.84 (0.67, 1.07)	0.85 (0.67, 1.07)
Iron and folic acid	0.85 (0.67, 1.07)	0.85 (0.67, 1.07)	0.85 (0.67, 1.07)	0.85 (0.67, 1.07)	0.85 (0.67, 1.08)

Values are IRR (95% CI).

The five models are identical with the exception of different cut offs to define low maternal preconception nutritional status. The prevalence of women with BMI cut offs of <18.5 kg/m^2^, <18.0 kg/m^2^, <17.5 kg/m^2^, <17.0 kg/m^2^ is 30.9%, 20.8%, 12.4% and 6.7%, respectively. The weight cut off of 43 kg is the lowest tertile in population.

Significant differences from two-tailed tests

*** p<0.001

** p<0.01

* p<0.05

+ p<0.1.

**Fig 2:**
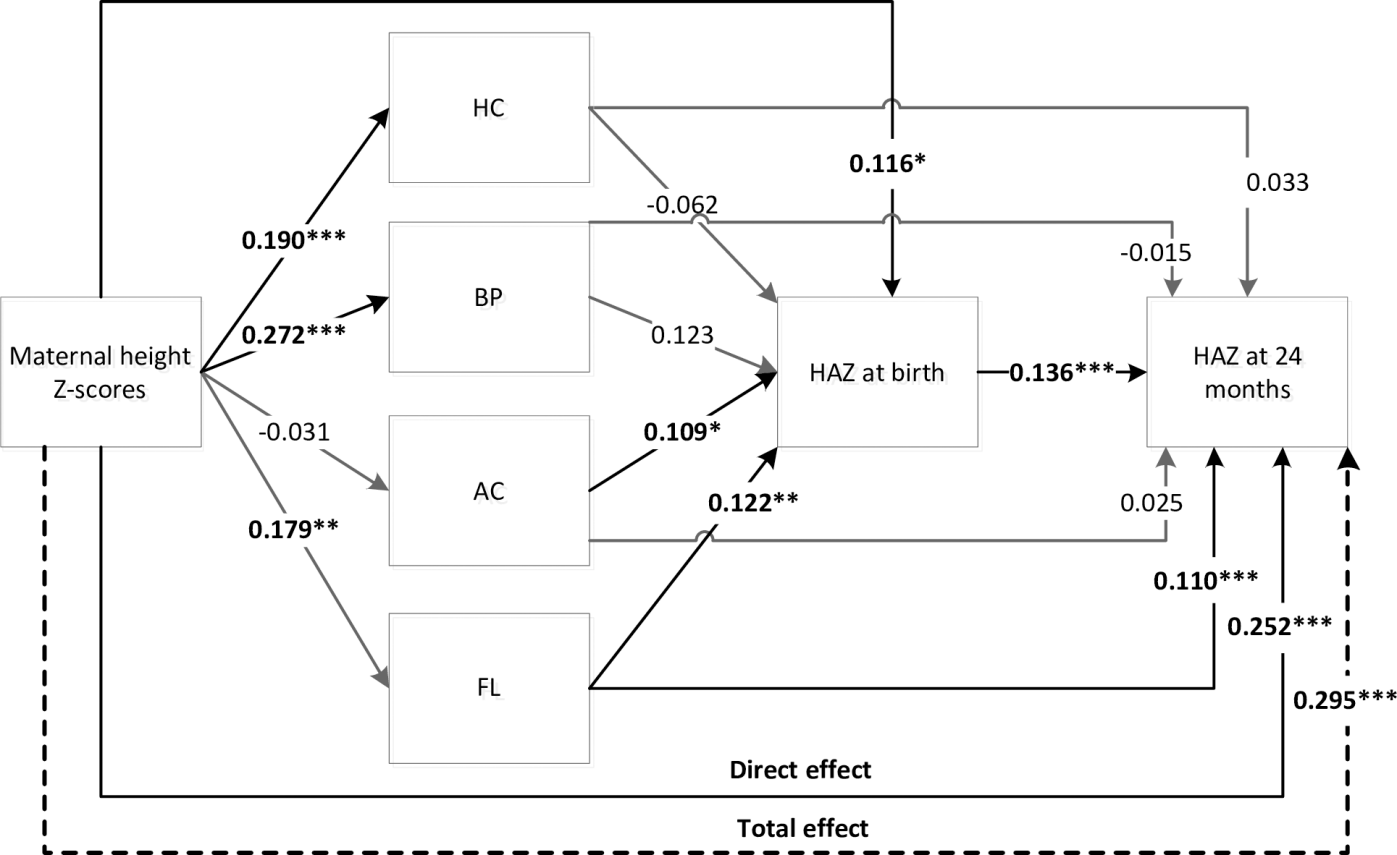
Direct and indirect effects of maternal height Z-scores on offspring HAZ at 2 years. Direct and indirect effects of maternal height Z-scores on offspring HAZ at 2 years. AC, abdomen circumference, BP, Biparietal diameter; FL, femoral length; HAZ, height for age Z-score; HC, head circumference. Significant differences from two-tailed tests: *** p<0.001, ** p<0.01, * p<0.05. Total effects: 0.30 SD*** (including direct 0.25 SD*** and indirect 0.04 SD** effects).

**Fig 3:**
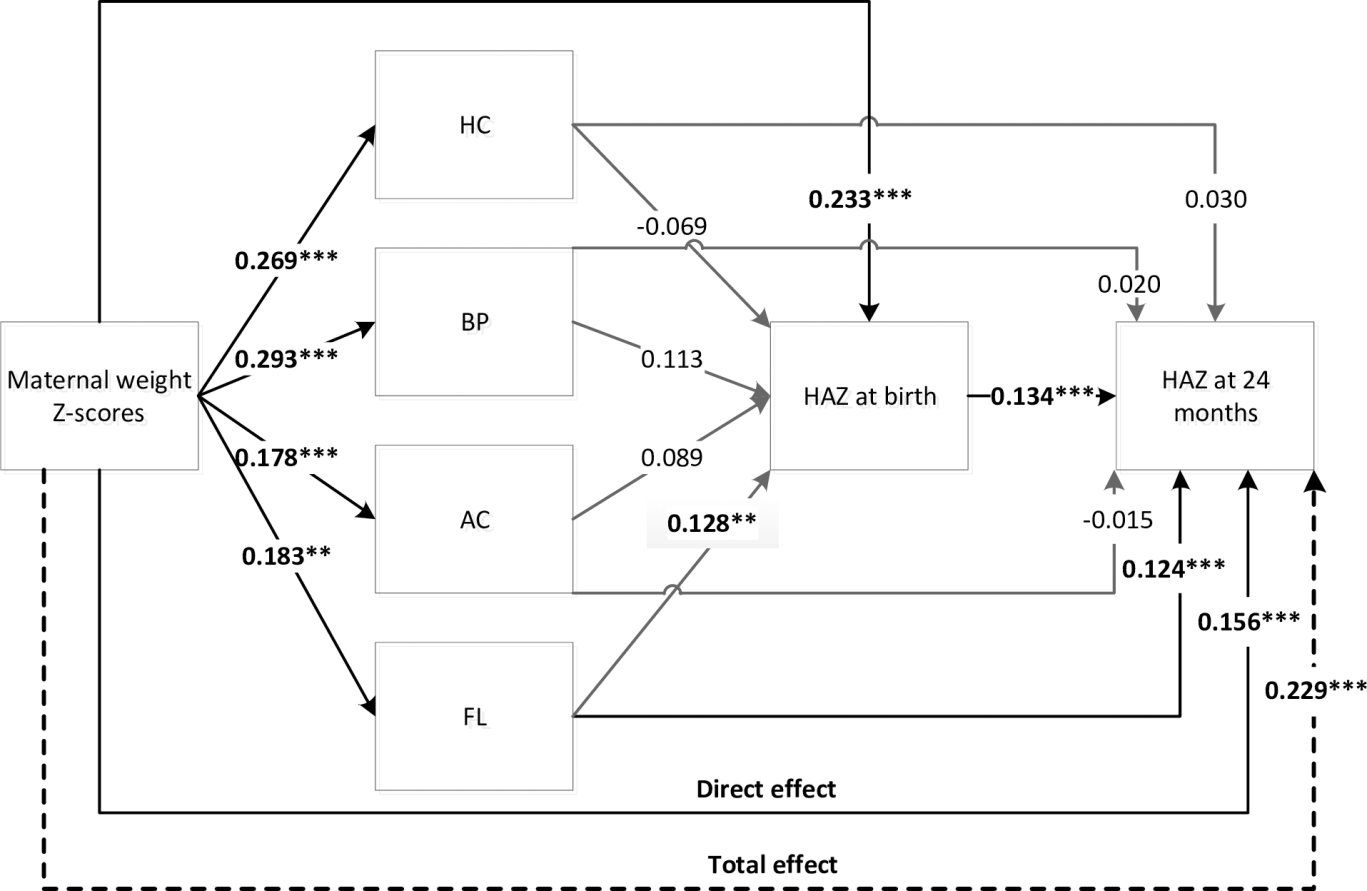
Direct and indirect effects of maternal weight Z-scores on offspring HAZ at 2 years. Direct and indirect effects of maternal weight Z-scores on offspring HAZ at 2 years. AC, abdomen circumference, BP, Biparietal diameter; FL, femoral length; HAZ, height for age Z-score; HC, head circumference. Significant differences from two-tailed tests: *** p<0.001, ** p<0.01, * p<0.05. Total effects: 0.23 SD*** (including direct 0.16 SD***and indirect 0.07 SD*** effects).

In the path analysis, PMNS was significantly and positively associated with child size at 2 years (**Figs 2 and 3**). For each 1 standard deviation (SD) increase in maternal height, offspring HAZ at 2 years increased by 0.30 SD (p < 0.001). A 1SD increase in maternal preconception weight was associated with an increase in HAZ at 2 years of 0.23 SD (p < 0.001). PMNS influenced child height at 2 years both directly and indirectly through fetal growth and attained size at birth (Figs 2 and 3).

## Discussion

This study demonstrates the importance of maternal preconception nutritional status on child linear growth across the first 1000 days. Women with a pre-pregnancy height <150 cm and weight < 43 kg, BMI <17.5 or BMI < 18 kg/m^2^ were at increased risk of having a stunted child at 2 years of age. The positive associations between pre-pregnancy height and weight z-scores and child HAZ at 2 years of age were explained partially through the influences on fetal growth and attained size at birth.

This study confirms and expands on prior research on the role of maternal nutrition on child growth. An analysis of data from 137 countries on key risk factors for child stunting reports an important role of maternal nutrition with 14.4% of the total stunting prevalence (6.4 million cases), being attributed collectively to maternal short stature, underweight, malaria and anemia [5]. In our study, maternal height was the preconception nutrition status indicator that was most strongly associated with child linear growth. Maternal height is an important indicator that may reflect a combination of the mother’s genetics and the nutritional and environmental factors that she experienced during her own childhood. In a study by Addo et al., the relative importance of maternal and paternal child growth on offspring birthweight were examined; and while both were associated with offspring size at birth there was a stronger matrilineal relationship [33]. These results imply that not only genetics but also the mother’s nutritional status during the 1^st^ 1000 days is critical for her child’s growth. The mother’s early nutritional status is reflective in her attained height in adulthood and the observed associations between maternal height and offspring growth and risk of stunting reflect the intergenerational transmission of malnutrition [34]. Prior analysis of 109 Demographic and Health Surveys, likewise reported a strong association between maternal height and child growth and mortality, with maternal height having twice the effect size as being in the lowest education category and 1.5 times the effect as being in the poorest wealth quintile [35]. In the COHORTS study examining five prospective birth cohorts (Brazil, Guatemala, India, the Philippines and South Africa), short women (<150.1cm) were 3 times more likely to have a stunted child [36] at 2 years. In our study, we were able to further examine both the direct and indirect effects of maternal height using prospective measures of fetal growth and size at birth.

Maternal pre-pregnancy weight and BMI are reflective of the mother’s current nutritional status and reserves available for fetal growth [37]. A recent review by King highlights the key underlying mechanisms and pathways by which maternal preconception nutrition may influence birth outcomes through influencing early placental and embryonic development, epigenetic effects as well as partitioning of nutrients between mother and baby [10]. In a meta-analysis of 34 studies, maternal preconception low BMI (<18.5 kg/m^2^) was associated with increased risk of preterm birth (RR 1.32; 95% CI 1.22–1.43) and SGA (RR 1.64; 95% CI 1.22–2.21) [9]. However, most of the prior studies have relied on mother’s recall of weight or on weight taken at first antenatal care visit, which may be biased, and reviews have noted the lack of high quality cohort studies [9, 11]. Further, few studies in low resource settings have prospectively followed children to examine the effect of preconception weight or BMI on child growth. In the current study, preconception weight was a significant predictor of child size at birth and linear growth at 2y. We also examined the association of lower gradations of maternal BMI and risk of child stunting using different cut offs to define low BMI (<18.5 kg/m^2^, <18.0 kg/m^2^, <17.5 kg/m^2^, <17.0 kg/m^2^). While the traditional cut-off to define maternal underweight (<18.5 kg/m^2^) was non-significant, women with a BMI of <18.0 kg/m^2^ or <17.5 kg/m^2^ were at increased risk of having a stunted child at 2 years of age. We were however unable to examine risk of more extreme levels of undernutrition as we had few women with a BMI <17.0 kg/m^2^. Overall, our findings point to a need for interventions to focus both on the early nutritional status of girls during the first 1000 days, as well as women’s nutrition in adulthood, before and during pregnancy, to impact offspring linear growth.

Key strengths of our study include the prospective study design and high follow-up rates with women who participated in the original PRECONCPT trial. The rich data on nutritional status that were collected prospectively across preconception and the first 1000 days allowed us to effectively use path analysis to examine the role of maternal preconception nutrition. We accounted for several covariates, however; residual confounding is possible. Future research would benefit from collecting additional detailed prospective information on biomarkers of nutritional status, infections (including both clinical and subclinical conditions such as environmental enteric dysfunction), inflammation and environmental exposures (i.e. mycotoxins; household air pollution) for both the mother and child to examine the direct and indirect effects and interactions with nutritional status across the lifecycle [5, 38–42]. Our study reports on the association between maternal preconception nutritional status at child height at 2 years (as a cumulative measure of child growth across the first 1000 days). Future conditional models would be beneficial for further understanding the importance of timing on rate of child growth across the lifecycle. The low prevalence of maternal overweight/obesity (5%) and excessive gestational weight gain (5.5%) in our population did not allow us to assess the role of maternal over nutrition for short and long-term child growth outcomes. Given the growing obesity epidemic across the globe, it will be important to replicate this study in diverse settings.

In conclusion, maternal preconception nutritional status influences both offspring linear growth and risk of stunting across the first 1000 days. Our results provide novel data on the role of maternal undernutrition before pregnancy on child linear growth at two years of age. These findings have implications on the importance of expanding the scope of strategies designed to reduce child stunting. Policies and programs focused only on women and children in the first 1000 days may miss the critical preconception period and thus full potential to benefit. Further research is required to expand these findings to inform the development of women’s nutrition programs that span the lifecycle and guide and inform policymakers.

## Supporting information

S1 Fig STROBE checklist. (DOC)
